# Insights on the effect of extracellular acidification on an HCN channel: A molecular dynamics study

**DOI:** 10.1371/journal.pone.0360058

**Published:** 2026-10-26

**Authors:** Somayeh Asgharpour, Michalis Lazaratos, Marc Spehr, Paolo Carloni

**Affiliations:** 1 Research Training Group 2416 MultiSenses – MultiScales, RWTH Aachen University, Aachen, Germany; 2 IAS-5/INM-9, Computational Biomedicine, Forschungszentrum, Jülich, Germany; 3 Department of Physics, Theoretical Molecular Biophysics Group, Freie Universität Berlin, Berlin, Germany; 4 Department of Chemosensation, Institute for Biology II, RWTH Aachen University, Aachen, Germany; University of Nova Gorica, SLOVENIA

## Abstract

Extracellular acidification can alter the structure and function of ion channels by neutralizing acidic residues exposed to the extracellular space. However, the relationship between protonation states and alterations in the structural dynamics of the channel is difficult to investigate by experiments. Here, we used atomistic simulations and graph-based algorithms to predict the H-bond pattern of the mouse hyperpolarization-activated cyclic nucleotide-gated (HCN2) channel, whose gating is facilitated by extracellular acidification. Our simulations revealed that E212 — a residue exposed to the extracellular space absent in human HCN channels — participates in a complex hydrogen-bond network with the transmembrane domain. These networks are partially disrupted at acidic pH levels, affecting the molecular interactions of the S4 helix in the channel’s voltage-sensing domain. On the basis of our findings, we suggest that hydrogen-bond network alterations under acidic extracellular conditions can affect channel’s gating.

## Introduction

Extracellular acidification may affect the voltage-dependent gating or conduction in ion channels, including potassium channels ([1–8]), sodium channels [9], proton channels [10], and chloride channels [11], along with non-selective channels such as hyperpolarization-activated cyclic nucleotide-gated (HCN) channels [12] and acid-sensing ion channels [13]. These properties can change due to altered protonation states of the histidines and acidic groups facing the extracellular space. This, in turn, can affect intermolecular hydrogen-bonds that originate at the extracellular site and extend into the protein interior. Thus, changes in the H-bond networks and long-distance water-mediated H-bond pathways might indirectly influence the gating process by strengthening or weakening key interactions that maintain the channel in a specific conformation. Here, we address this issue by performing molecular dynamics (MD) simulations and graph-based hydrogen-bond analysis ([14,15]) on HCN channel type 2 (HCN2) from mouse. This channel, along with HCN type 4 (HCN4), exhibits a depolarizing shift in their activation voltage in response to extracellular acidification [12], which facilitates the gating mechanism [16].

HCN channels consist of four identical subunits (Fig 1a). Each subunit includes six transmembrane α helices (S1-S6), the *pore domain* and the *HCN domain*, which is a bundle of three helices specific to these channels [17]. The cytoplasmic domain contains the cyclic nucleotide binding domain (CNBD), where cyclic nucleotides can bind [17], shown in Fig 1a (colored green). Gating of this channel and conduction of ions depend on the collective motion of helices S1-S4 (*voltage sensing domain, VSD)* and the pore (Fig 1c).

**Fig 1:**
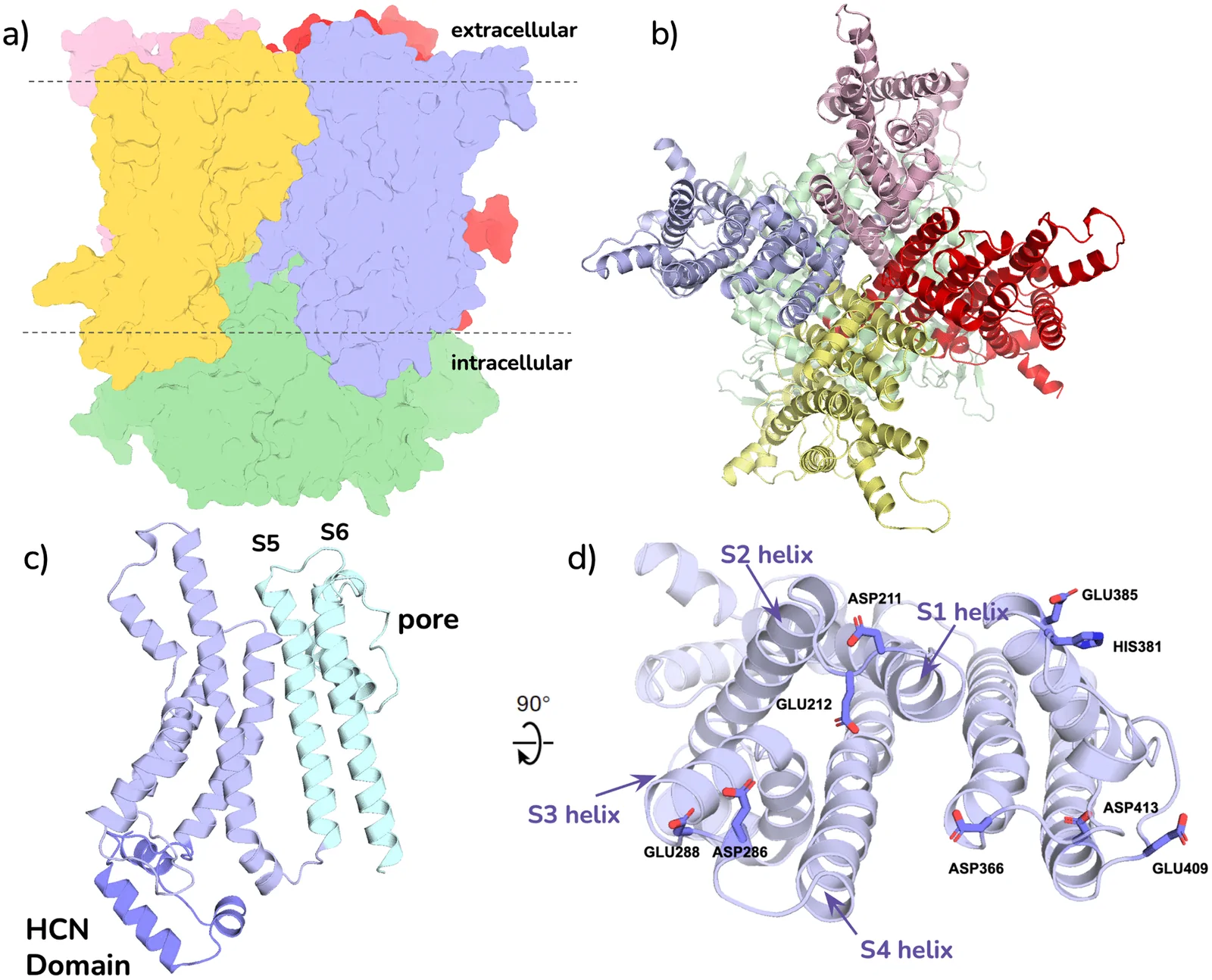
Mouse HCN2 structure. This structure was predicted based on the highly similar human HCN1 cryo-EM structure (PDBid: 5U6O; [18]), which shares 91% sequence identity. The protein is made up of four subunits and one cyclic nucleotide-binding (CNB) domain. (a) Structural model of the protein. Each of the four subunits is shown in a different color: the yellow and the blue ones point towards the viewer. The CNB domain is colored in green. (b) Orientation of the channel showing the pore and extracellular site. (c) Structure of a single subunit. This consists of the transmembrane domain (made up of α-helices S1–S6 and the pore), along with the HCN domain. (d) Titratable amino acids on the extracellular site of one subunit.

For the pore to open, the S4 helix must undergo a significant conformational change [19], associated with a rearrangement of salt bridges and H-bond interactions between the nine basic residues of S4 (Fig 2) and the acidic residues on helices S2 and S3 [20,21]. As seen in Fig 2, the S4 helix moves downwards by approximately two helical turns and tilts almost $80^{\circ }$, this breaks many salt bridges between positive charges on helix S4 and countercharges on S1-S2-S3 helices, including K303-E212, R309-D275, and R312-D225. The main question addressed here is whether this network of interactions could change upon extracellular acidification in the resting state, and if so, whether it provides insight into how these changes might affect the gating process. To address this issue, we predict the conformational landscape of the resting state by performing two MD simulations at pH 3.0 and pH 7.0.

**Fig 2:**
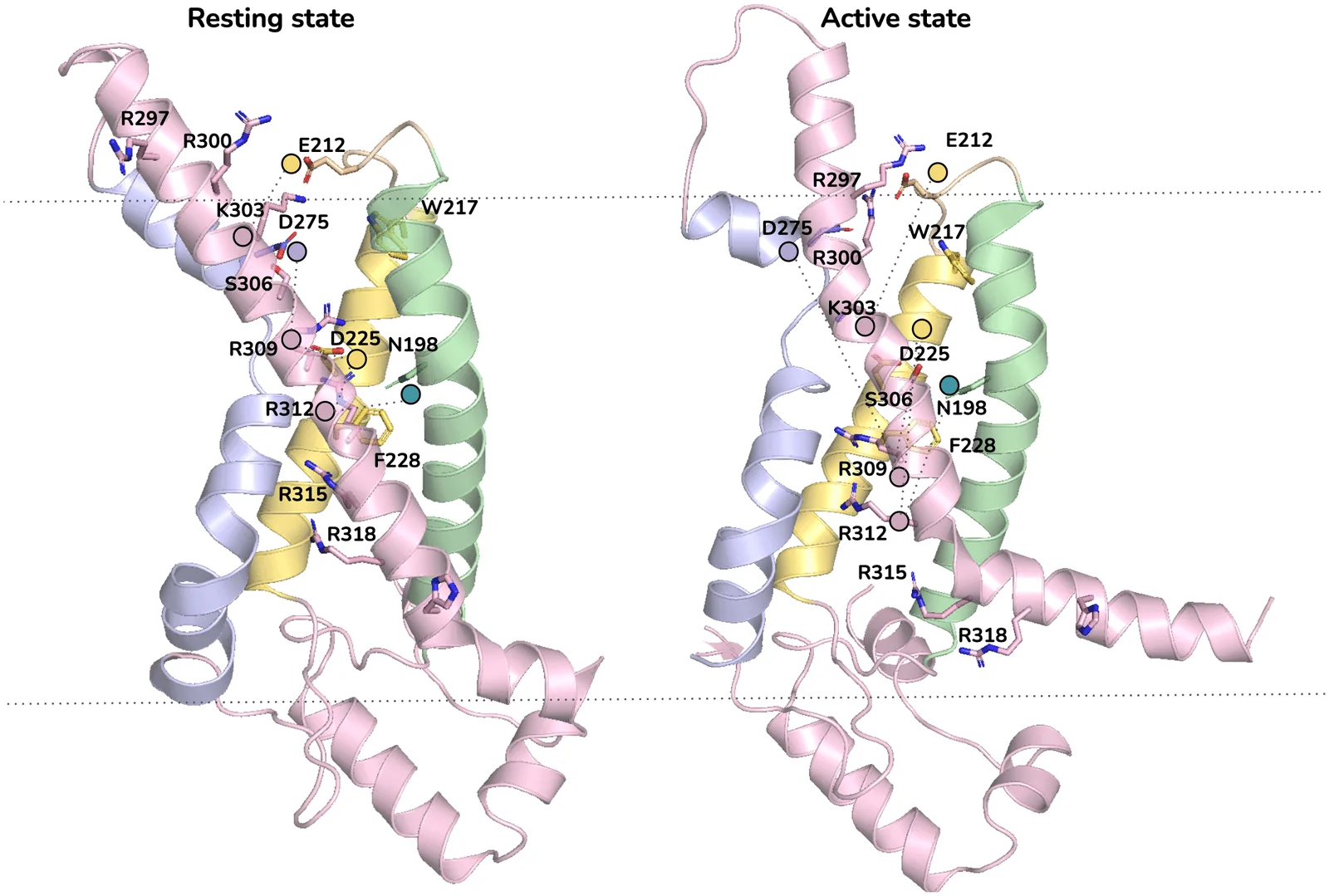
Mouse HCN2 channel voltage-sensing domain structure in resting and open states. These structures were predicted based on the highly similar human HCN1 cryo-EM structures (PDBid: 5U6O, 6UQF; [18,19]). The S4 helix moves downward by two helical turns and tilts by about $80^{\circ }$.

## Materials and methods

### Structural models

The cryo-EM structures of human HCN type 1, 2, and 4 channels (HCN1, HCN2, and HCN4) with VSD in active and resting states have been solved ([17,19]). A model of the mouse HCN2 channel (UniProtKB – O88703 (HCN2-MOUSE); https://www.uniprot.org/uniprot/O88703) including all domains (VSD, CNBD, HCN domain, and pore domain) was generated by homology modeling using Swissmodel [22]. The structure of human HCN1 (PDBid: 5U6O) in its resting state was used as the template. The models and the templates are highly identical sharing 91% sequence identity (Fig 3). In the voltage-sensing domain (VSD), sequence identity is 100% for S4 and 82%, 70%, and 79% for helices S1–S3, respectively.

**Fig 3:**
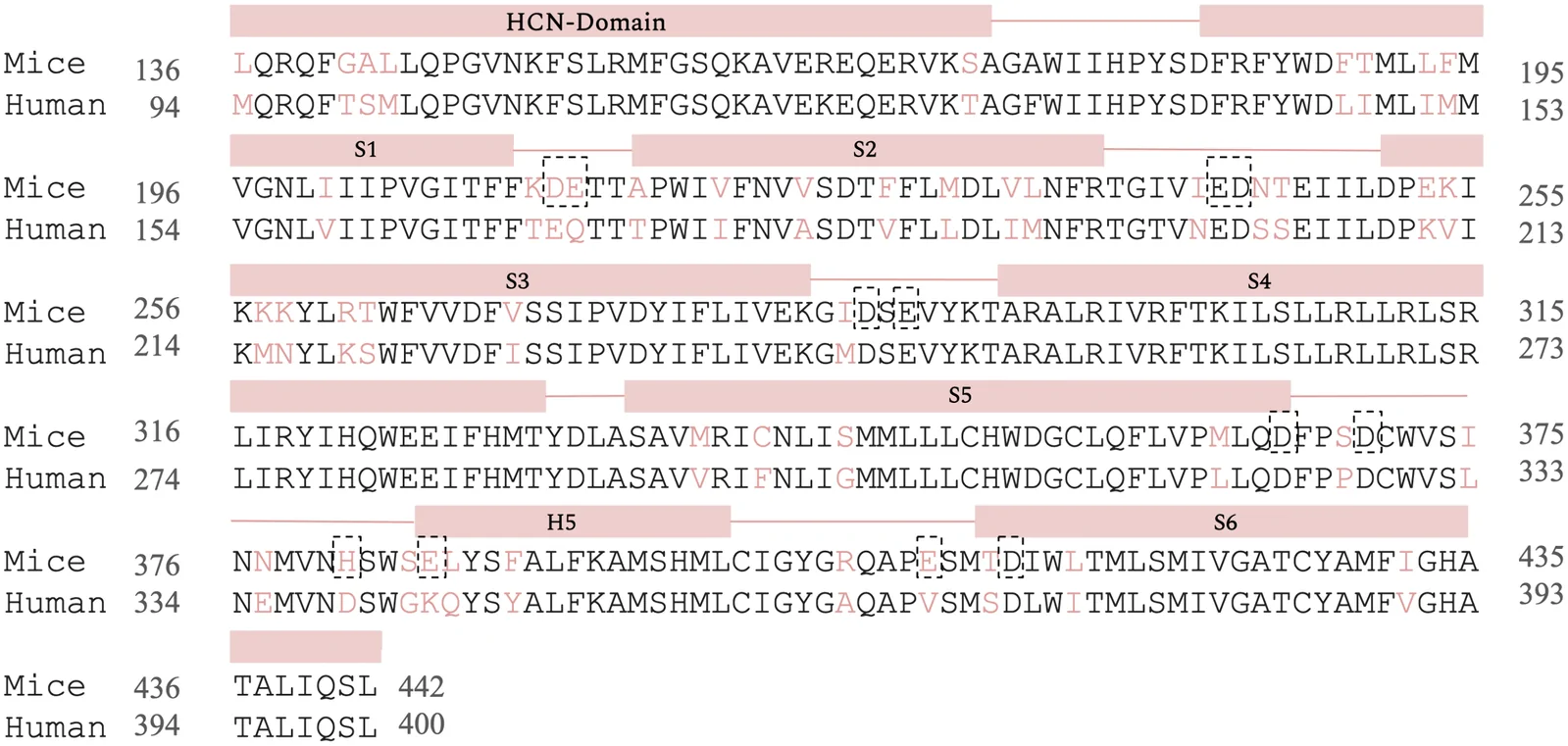
Sequence alignment between human HCN1 (PDBid: 5U6O) and Mouse HCN2, dashed rectangles indicate amino acids undergoing acidification and colored amino acids correspond to those which that are different across Mouse and Human channels.

### Simulation set-up

The systems were embedded in a hydrated bilayer of palmitoyl-oleoyl-sn-glycero-phosphocholine (POPC) lipids using the CHARMM-GUI server [23]. Water and ions were added to fill up a box of 130 Å, 130 Å, 170 Å edges. They contained ≈ 70,000 water molecules. At physiological pH, the system contained 190 K^+^ and 198 Cl^–^ ions, while the one at pH 3.0 included 190 K^+^ and 238 Cl^–^ ions. Both systems were neutralized in ≈ 150mM KCl. Each system consisted of ≈ 300,000 atoms. The $pK_{a}$ values of the extracellular titratable residues (H381, E385, D366, E409, D413, D211, E212, D286, and E288) were calculated using PROPKA (S1 Table) ([24,25]) modules in Maestro Schrödinger Software [26] {For the choice of the residues to be considered as titratable by external acidification, see SI}. Based on these values, the protonation states were set at physiological pH (where all the residues turned out to be ionized except H381 that was neutral) and at pH 3.0 (where all acidic residues were neutral, whereas H381 was positively charged).

### MD simulations

We used the CHARMM36 force field for protein, lipids, and ions [27] and the TIP3P water model [28]. Long-range interactions were evaluated using the particle-mesh Ewald summation (PME) with a cutoff of 12 Å for the real-space component. The Lennard-Jones interactions were truncated at 12 Å with an atom-based force-switching function, effective at 10 Å. The integration time step was set to 2 fs. All bonds were constrained by the LINCS algorithm [29]. The systems were minimized after 2,000 steps of steepest descent. Each system was then equilibrated for 1 ns in the NVT ensemble (T = 300K). This was followed by a 1 ns-long equilibration in the NPT ensemble (P = 1 atm). We used Berendsen’s thermostat and barostat [30]. Two 1.2 μs-long NPT simulations were performed using the Nosé-Hoover thermostat [31] with an effective relaxation time of 1 ps, a semi-isotropic Parrinello-Rahman barostat [32] and a pressure coupling effective every 5 ps with compressibility of $4.5\times 10^{-5}$. All MD simulations were performed using the GROMACS-2020 package [33]. All trajectories of Cartesian coordinates in the XTC format are deposited in a dataset MDposit storage interface at https://irb-dev.mddbr.eu/#/browse?search=Somayeh. The analysis of the MD trajectory was carried out for the last 800 ns of the dynamics (equilibrated part, see Results section).

### Graph-based analyses

Graphs of water-mediated and/or direct H-bond networks were generated using the Bridge code [14] and the Bridge2 graphical user interface [34]. An H-bond is assumed to be formed when the distance between the H-bond donor and acceptor is within 3.5 Å, and the H-bond angle is within 60 degrees. An occupancy of 100% means that an H-bond is formed throughout the entire trajectory segment used for data analysis. Here, we computed these graphs using 8,000 equally spaced coordinate snapshots with time intervals of 100 ps for the equilibrated trajectory. H-bonds with high occupancies > 50% were included in the analysis. Additional details are provided in the SI.

## Results and discussion

Each of the four subunits, at the extracellular site, contains one histidine, four aspartates, and four glutamate residues (H381, E385, D366, E409, D413, D211, E212, D286, and E288) as titratable residues. At physiological pH, the histidine residue is assumed to be neutral and protonated on its δ nitrogen, while the acidic residues are assumed to be negatively charged. At pH 3.0 (in extracellular medium) these residues are all protonated, while H381 is positively charged (see S1 Table). Two 1.2 μs trajectories were generated, including the channel, membrane, solvent, and ions, at physiological pH and at pH 3.0 under extracellular acidification. The channels’ structural determinants were well maintained during dynamics (see SI for details). Both simulations converged after 0.4 μs, as indicated by the RMSD analysis (S1 Fig). Here, we present the results for one subunit. Results for the other three subunits are given in SI. We observe the following pathways at physiological pH: **path1**: E212-Y276-D275-R309-D225-R312, with an occupancy threshold of 80% (Fig 4a). **path2**: E212-D275-R312 with an occupancy threshold of 53% (Fig 4b), and **path3**: E212-W217-R309-D225-R312 with an occupancy threshold of 55% (Fig 4c).

**Fig 4:**
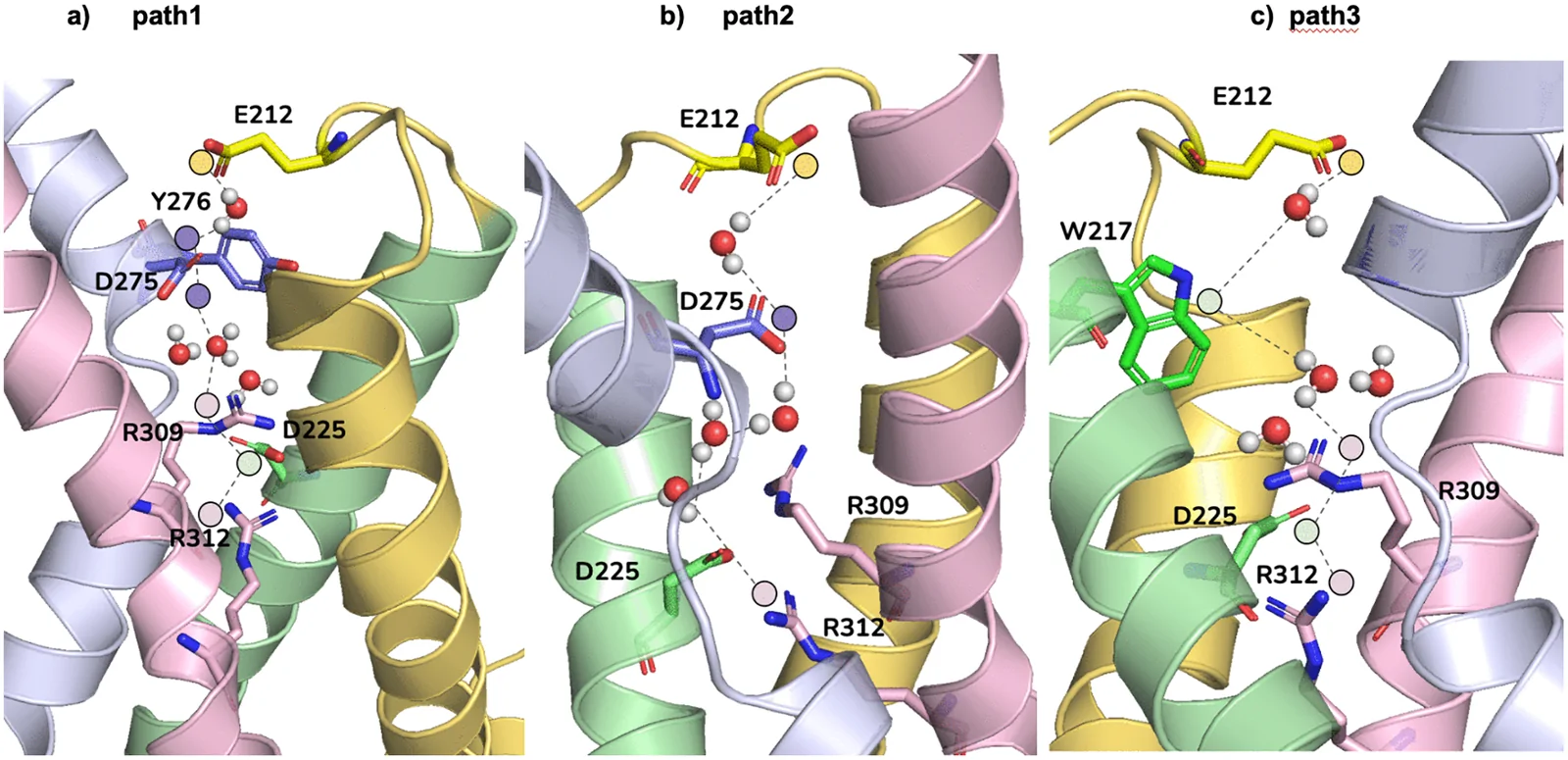
Water-mediated pathways that connect E212 at the extracellular domain to R312 of helix S4. Three different pathways are shown.

These networks involve few water molecules. E212 is locked in a salt bridge with K303 (100% occupancy, see Fig 5a). These three paths, in which E212 forms water- mediated H-bond interactions either through Y276, or D275, or W217 (Fig 5a, 5b), may help maintain the channel in its resting state.

**Fig 5:**
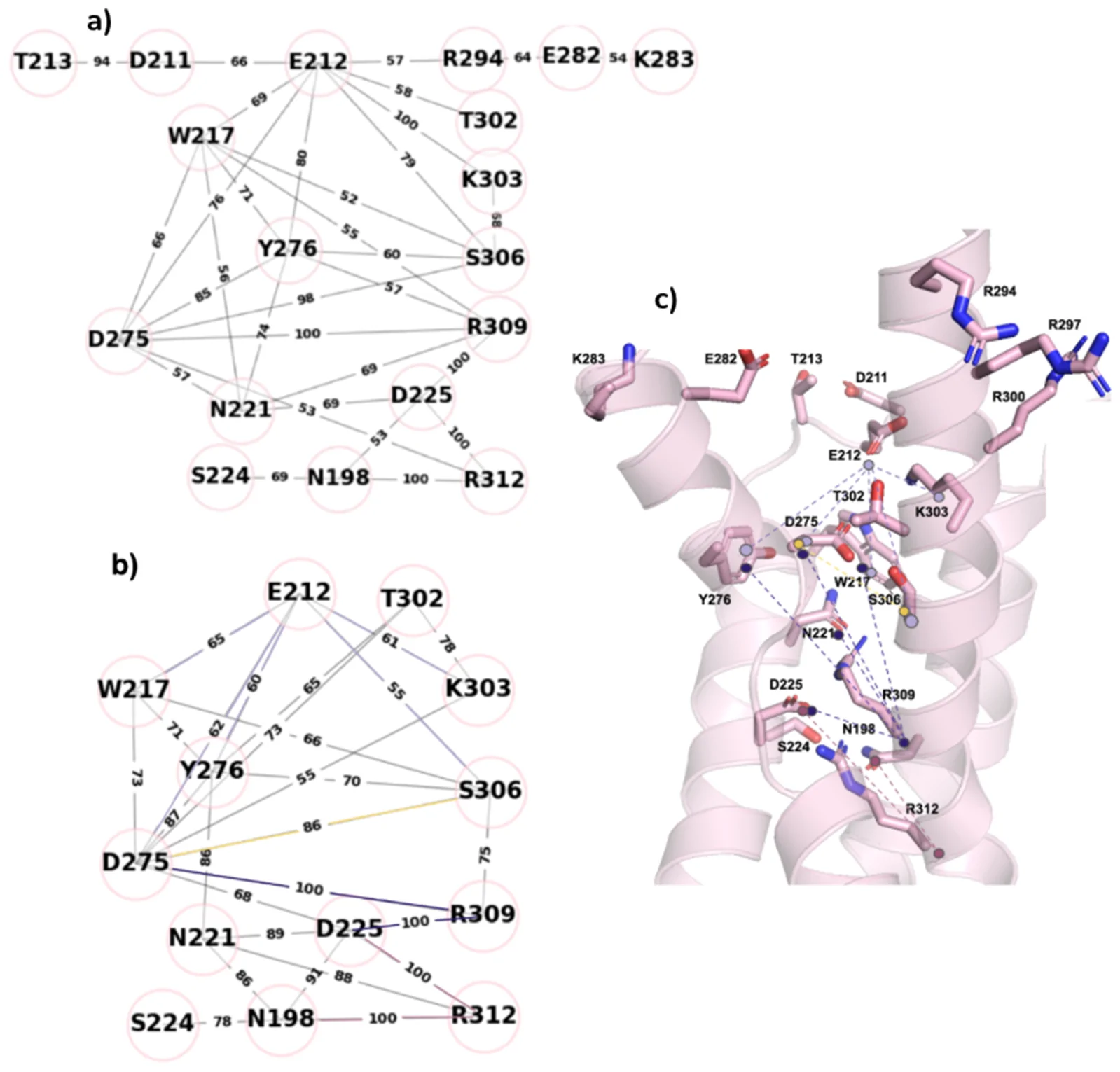
H-bond network connecting E212 to R312 in the VSD. The most persistent interactions are shown as colored lines. The number on the line represents the percentage of persistency. The H-bond interactions can be direct or water-mediated. **(a)** At pH 7, this network is very persistent. **(b)** At pH 3, the network is partly disrupted. **(c)** Structure of the H-bond network. Key amino acids are shown here with the same color as in the network.

At pH 3.0 (Fig 5b), the occupancy of the E212 - K303 interactions is reduced from 100% to 60% and, as a result, this residue is more flexible. Protonation of E212 causes the loss of the salt bridge between the two residues. As a result, only **path1** is still present. In the other two, the water-mediated H-bond interactions between E212 with either D275 on **path2** or W217 on **path3** are lost.

The H-bond networks of the other extracellular residues do not indicate a significant change upon acidification. The other two subunits show results similar to the first subunit. However, in subunit C, the residue T213 replaces E212 in the network with the VSD (see Supplementary Information). The difference observed between the subunits may be expected, especially considering that it involves individual residues located in loops, such as T213 and E212. It may arise from the stochastic nature of the trajectory generation, including the initial velocity assignment. Importantly, this difference does not affect the qualitative behavior of the systems or the overall conclusions of the study. The simulations indeed are well converged, exhibiting fluctuating RMSD values after an equilibration phase of 400 ns.

Measuring gating properties of E212 mutants, which may disrupt this H-bond pattern (e.g., E212A), could confirm the proposed role of this residue in mouse HCN2 gating upon extracellular acidification. Interestingly, E212 is also conserved in the mouse HCN4 isoform, suggesting that this residue may play a similar role in response to extracellular acidification of this channel [12]. However, this key residue is not conserved in human channels: A sequence comparison reveals a notable species-dependent difference at the position identified in this study. Although the mouse channel contains a KDE motif, the corresponding region in the human HCN1 channel (which exhibits a positive shift in voltage sensitivity upon extracellular mutation, as reported by Mckenzie et al., 2022 [35]) is TEQ. While the pH dependence of human HCN1 has not yet been established, we postulate here that the experimentally observed over-activation of the channel in the TEQ → TAQ mutant may be caused, at least in part, by changes in the intramolecular H-bond network. Therefore, the present findings help provide insight into the mechanism that contributes to pH sensitivity in the mouse channel, while simultaneously highlighting the importance of this motif for intramolecular hydrogen bonds of the protein in the human HCN1 channel.

We emphasize that our results are qualitative and limited to the resting state. The observed shift in voltage-dependent gating could originate from a disruption of the resting state relative to the activated state or from stabilization of the activated state relative to the resting state. Free energy calculations of the gating process at different pH values and/or simulations of the open state could clarify this.

Another limitation of the present study is the use of fixed protonation states. Constant-pH MD simulations, which allow protonation states to vary during the simulation [36], are more computationally expensive than standard MD. They are particularly important at pH values near the pKa of acidic residues, which is not the case here, since we focus on physiological and highly acidic pH levels. In both conditions, the protonation states of the acidic residues are unambiguous.

## Conclusion

Most MD studies of HCN channels have focused on physiological pH, providing insights on ion permeation and selectivity in the human HCN1 [37] and HCN4 channel [38], also in the presence of a blocker such as ivabradine ([39,40]). Here we used MD simulation in an attempt to gain insights on the impact of extracellular acidification on the channels’ function. Based on our calculations, we suggest that acidification indirectly affects the gating mechanism by altering the H-bond networks from the extracellular space to the VSD ([12,16]). Specifically, the long water-mediated hydrogen-bond network between R312 and E212 weakens at low pH in most subunits of the channel (Fig 5). At pH 3.0 the S4 helix may become more “loose,” which might contribute to downward motion toward the cytoplasm — a hallmark of the gating process [19]. This could help explain the change in gating behavior observed with extracellular acidification [12].

## Supporting information

S1 TableEstimated $pK_{a}$ values of ionizable residues located in the extracellular region of the channel.We used the Propka 3.1 module ([24,25]) in Maestro Schrödinger software [26] to obtain estimated values $pK_{a}$ of ionizable residues at the extracellular site in the resting state of the channel.(DOCX)

S1 Fig The Root-mean-square-deviations (RMSD) of the voltage-sensing domain (VSD) helices and connecting loops. The RMSD was calculated for the four VSD helices, both with and without the connecting loops. The RMSD values range from 0 to 5 Å and stabilize after approximately 400 ns. RMSD profiles are shown at physiological pH (pH 7.0; upper panel) and pH 3.0 (lower panel), together with smoothed histograms of the RMSD distributions throughout the trajectory.
(TIF)

S2 Fig The root-mean-square-fluctuations (RMSF) of the voltage-sensing domain (VSD) and connecting loops. The RMSF profiles are shown for simulations at pH 7.0 (light colors) and pH 3.0 (dark colors). The largest fluctuations occur in the loops connecting the S2 and S3 helices. The relative RMSF values at the two pH conditions vary among subunits. In subunit D, the RMSF is higher at pH 7.0 than at pH 3.0, whereas the other subunits show similar or lower RMSF values at pH 7.0. The loop connecting S2 and S3 exhibits greater fluctuations at lower pH in subunit A, lower fluctuations in subunit C, and similar fluctuations in subunits B and D. In subunit C, E212 exhibits a higher RMSF in its deprotonated state, whereas the corresponding values in the other subunits are similar or lower.
(TIF)

S3 Fig Hydrogen-bond networks in subunit A under different water-wire criteria. The hydrogen-bond network was analyzed using four approaches that varied the number of water molecules allowed to participate in water wires. Direct H-bonds were analyzed alongside networks incorporating water wires containing one, two, or three water molecules. Including water wires expands the H-bond network, particularly within the VSD, compared to the case of only considering direct hydrogen bonds.
(TIF)

S4 Fig Hydrogen-bond networks connecting extracellular residues to the VSD in subunits B, C, and D. Hydrogen-bond networks connecting E212 to R312 are shown using a 50% occupancy threshold, following the analysis performed for subunit A (see main text). The networks contain fewer H-bonds and exhibit lower occupancies at pH 3.0. These observations are consistent with the possibility that protonation of E212 affects channel gating by weakening its hydrogen-bond connectivity with the VSD. In subunit C, T213 forms part of the hydrogen-bond network connecting the extracellular region to the VSD in place of E212. This asymmetry is associated with an initially upward conformation of E212, which interacts with water molecules for more than 80% of the simulation time.
(TIF)
