# Supplementary material for: Insights on the effect of extracellular acidification on an HCN channel: A molecular dynamics study
Source: PLoS One. 2026 Oct 26;21(10):e0360058. doi: 10.1371/journal.pone.0360058 (PMC13704849; doi:10.1371/journal.pone.0360058)
Supplement: Supplementary file 1 [file pone.0360058.s001.docx]

Computational Details

Table 1. **Estimated *pK_a_* values of ionizable residues located in the extracellular region of the channel. We used the Propka 3.1 module ([24], [25]) in Maestro Schrödinger software [26] to obtain estimated values *pK_a_* of ionizable residues at the extracellular site in the resting state of the channel.**

| **Residue** | ***pK_a_*** |
| --- | --- |
| ASP 211 | 3.1 |
| GLU 212 | 5.3 |
| ASP 286 | 3.9 |
| GLU 288 | 4.5 |
| ASP 366 | 4.0 |
| ASP 370 | 4.0 |
| GLU 385 | 3.0 |
| GLU 409 | 4.9 |
| ASP 413 | 5.3 |
